# Commissioned reports in Swedish healthcare governance – descriptive mapping and a content analysis

**DOI:** 10.1186/s12913-022-09006-x

**Published:** 2022-12-30

**Authors:** Anna T. Höglund, Erica Falkenström, Stefan Svallfors

**Affiliations:** 1 Centre for Research Ethics and Bioethics, Department of Public Health and Caring Sciences, Centre for Research Ethics and Bioethics, Uppsala University, Box 564, SE-751 22 Uppsala, Sweden; 2 Institute for Future Studies, Stockholm, Sweden

**Keywords:** Commissioned reports, Healthcare governance, Document analysis, Equity in care, Knowledge-based decision-making, Sweden

## Abstract

**Background:**

In order to support decisions regarding governance, organization and control models of the healthcare system, the Swedish government, as well as regional-level agencies, regularly commissions expert reports that are supposed to form the basis for decisions on new steering forms in healthcare.

**Aim:**

The aim of this study was a) to perform a descriptive mapping of commissioned reports on Swedish healthcare governance and b) to pursue an in-depth content analysis of a strategic sample of such reports.

**Method:**

Initially, 106 reports from both national and regional levels were gathered and analysed. A matrix was constructed, consisting of questions on who had commissioned the report, who had produced it, what problems the report set out to solve and what solutions were suggested. Further, questions were posed on whether the report was research-based and whether ethical assumptions and arguments were presented. Thereafter, a strategic sample of 36 reports was selected for an in-depth analysis, using inductive content analysis.

**Results:**

The descriptive mapping showed that the aim of the analysed reports differed in form and content, and that they varied from giving an overview and investigating effects and consequences of new control models to more concrete goals, such as suggesting improvement measures. Academic experts involved in creating the reports often represented economics or business studies. The content analysis revealed examples of standardization in care, characterized by requirements to follow national guidelines, but also examples of requests for increased respect for professionals’ competence and experience. Further, the analysis showed how the definition of equity in care had changed, from a focus on equity in access to care in the reports produced in the 1990s to an emphasis of arguments for geographical sameness and equity in quality of care in the later reports.

**Discussion:**

Two dominant trends were identified in the material, namely increased standardization and arguments for trust in the system. The great number of reports implies that the system risks requesting more information than it can handle and result in documents where the same message is recurrently repeated or create conflicts of interest and value tensions between different suggestions.

**Conclusion:**

Commissioned reports can have substantial consequences for new reforms of management practices in healthcare. It is therefore important to investigate them critically. The results of our investigation may contribute to a more comprehensive and adequate model for acquiring and using expert reports regarding healthcare governance, both in Sweden and in similar healthcare systems.

## Background

An established ideal of democracy is that political decisions should be based on enlightened understanding [1]. This means, that it is not democratically acceptable to neglect information that is relevant to the decision to be made. In line with this, decision-makers should not demand only a certain kind of knowledge or consciously use knowledge that is produced selectively. If they do so, the search for knowledge, for example, in public inquiries, takes the form of an empty ritual and can become a manipulative tool that only aims to give legitimacy to the decisions made [2, 3].

The Swedish healthcare system is an example of an organization that aspires to democratic legitimacy through such knowledge-based decision-making. There is extensive previous research concerning the role of knowledge and information in relation to decision-making and management in organizations. A recurring conclusion from this research is that organizations often collect more information than they can handle, and that the information that is collected often has little relevance to the decisions made. In spite of this, the collection of information has important functions for the organizations in question, since it lends an appearance of rationality to decision-making [4, 5].

The Swedish government, as well as regional-level agencies, regularly commissions expert reports that are supposed to form the basis for later decisions regarding governance, organization and control models in the healthcare system. In an ongoing project, we have conducted an analysis of the quantity and content of such reports commissioned by national and regional authorities. We investigate the kind of knowledge that is requested at the political and administrative levels of the healthcare system and the knowledge that is actually produced through such reports. We have further investigated the motives behind the commissioning of reports and their later consequences. We approach the issue of knowledge in healthcare governance from a slightly different angle than previous research, as we focus on the actual reports that have been intentionally commissioned with a stated goal to improve governance and organization of the healthcare system.

When it comes to management practices, studies show that organizations to a large extent tend to follow organizational fashions [6]. Earlier research has focused on knowledge-based decision-making [7–12]. It stems from the well-established research field of knowledge management [13]. The attention here is on creating more efficient systems for collecting, analysing and disseminating knowledge. Further, the aim is to gain knowledge support throughout organizations in order to increase their efficiency [14, 15].

Over the course of several decades, the organization of healthcare in Sweden, as in many other countries, has changed. Through a number of reforms, the dominant logic has shifted from professional dominance and political control towards managerial control through market mechanisms. A crucial motive behind the market reforms was to increase cost-efficiency. However, a central part of the Swedish welfare system, including healthcare, is mainly publicly funded and, according to Swedish law, all healthcare provision should be underpinned by ethical values and norms. For example, the Swedish Healthcare Act [16] prescribes that healthcare should be provided according to needs and with respect for each person’s human dignity. The goal is equity in health for the whole population. The responsibility for healthcare governance is divided between national and local levels. Self-governing county councils are responsible for the financing and provision of healthcare in 21 regions.

Among the ethical underpinnings of Swedish healthcare are the guidelines for priority setting prescribed by the National Board of Health and Welfare and decided by the Swedish Parliament in 1997. These guidelines form a platform consisting of three ethical principles to inform priority setting on the national, political and clinical levels. The principles, prescribed in descending order of importance, are *the principle of human dignity, the principle of need and solidarity* and *the principle of cost-efficiency* [17]. According to the principle of human dignity, all human beings are equal in value, regardless of characteristics and functions in society. The principle of need and solidarity holds that healthcare resources should be provided to the patient most in need and that special attention should be given to persons with limited autonomy. The cost-efficiency principle, finally, implies that a reasonable relation between cost and effect should be aimed for in all healthcare provision. In order to reach the Swedish goal of equity in health, political governance and the design of the healthcare system and its functions are of great importance, as is the choice of control models in the system.

The results presented in this article are part of a larger study. We present a descriptive mapping of commissioned reports from 1993 to 2020, as well as a content analysis focusing on trends and differences in a strategic sample of commissioned reports. Our goal is to contribute empirically grounded research concerning the reports that are regularly commissioned and produced in the Swedish healthcare system.

Against this background the aim of this study was a) to perform a descriptive mapping of commissioned reports on Swedish healthcare governance and b) to pursue an in-depth content analysis of a strategic sample of such reports. In the descriptive mapping, the following research questions were investigated:

- Which individual(s) or institution has commissioned the studied reports?
- Who has produced it?
- What problems does the report set out to solve and what solutions are suggested?
- Is the report research-based?
- Are ethical assumptions and arguments presented?

In the content analysis, the following research questions were investigated:

- What characterizes the content of the studied reports?
- What different forms of governance or control models do they represent?
- What trends and differences can be found in the reports, and are they compatible or do they represent conflicting ideals and values?

Our investigation is primarily empirically driven, where the strength of the investigation lies in the great number of reports that have been gone through thoroughly. However, the results will be discussed in relation to different theoretical concepts and theories based in ethics and organizational theory.

## Method

### Material

Initially, 106 reports from both national and regional levels commissioned between 1993 and 2020 were gathered and analysed. The time span 1993–2020 was chosen because it represents a time of intense debate on healthcare governance in Sweden. Apart from that, we aimed to gather a broad and rich assortment of material. Inclusion criteria were that the reports should deal with healthcare governance and be commissioned by the healthcare system in a broad sense. That means that they could be commissioned by, for example, the Ministry of Social Affairs or the National Board of Health and Welfare. Apart from that, we also included regional reports, in order to get an even broader range of material. We do not claim to have included every single report produced during the selected time span, but we have included all reports we were able to find that fit the inclusion criteria.

Thereafter, a strategic sample of 36 reports was selected for an in-depth analysis. The strategic sample was based on the aim of analysing a broad variety of reports, although it was limited to a quantity that would be manageable for our analysis. It included national reports, regional reports, reports written by researchers and reports written by experts, civil servants and political representatives. Among these, 32 were national documents and four were local. The national reports were of several types, such as public inquiries, commissioned expert reports and consultant reports. The local documents consisted of two reports from the Stockholm Region and two from the Norrbotten Region. A list of the 36 reports that were analysed in-depth can be found in Table 1.

**Table 1:** Overview of the 36 in-depth analysed reports, chronologically listed

Year	Commissioner	Title
1993	The Government of Sweden	SOU 1993:38: *Hälso- och sjukvården i framtiden – tre modeller* (Future health care—Three models)
1999	The Government of Sweden	SOU 1999:66: *God vård på lika villkor? Om statens styrning av hälso- och sjukvården* (Good health on equal terms? On public governance in the health care sector)
2001	The Government of Sweden	SOU 2001:8: *Prioriteringar i vården. Perspektiv för politiker, profession och medborgare* (Priority-setting in health care. Perspectives for politicians, professionals and citizens)
2005	The Swedish Agency for Public Management	*Modeller för styrning: Förslag om hur staten kan styra kommuner och landsting* (Models of governance: Suggestions of public governance models for municipalities and county councils)
2006	The Swedish Association of Local Authorities and Regions (SALAR)	*Kunskapsbaserad ledning, styrning och utveckling inom hälso- och sjukvården* (Knowledge-based management, governance and development in health care)
2009	The Stockholm Region	*Framtidens hälso- och sjukvård* (Future health care)
2010	SALAR	*Ett nytt tänk. Öppna jämförelser i hälso- och sjukvårdens ledning, styrning och kvalitetsarbete* (A new way of thinking. Open comparisons in management, governance and quality work in health care)
2010	The Expert Group on Public Economics	*Värden i vården* (Values in health care)
2011	The Stockholm Region	*Styrformer och arbetsförhållanden inom vård och omsorg* (Management forms and working conditions in the health care sector)
2011	The Norrbotten Region	*Unika utmaningar och unika möjligheter* (Unique challenges and unique possibilities)
2012	The Government of Sweden	SOU 2012:33: *Gör det enklare*! (Make it easier!)
2012	The Government of Sweden	SOU 2012:33a: *Den mångfaldiga styrningen i hälso- och sjukvården* (The diversity of management forms in health care)
2012	The Government of Sweden	SOU 2012:33b: *Med fokus på prevention och jämlikhet* Focusing on prevention and equality)
2012	The Government of Sweden	SOU 2012:33c: *Gör det enklare. Kunskapsunderlag* (Make it easier. Knowledge base)
2012	The National Board of Health and Welfare	*Styrning med förhinder* (Management with prevention)
2012	The National Board of Health and Welfare	*Kunskapsstyrning för ledning och policyarbete* (Knowledge-based governance for management and policy work)
2014	SALAR	*Intermountain Healthcare. Styrning för kvalitet i ett högpresterande system* (Intermountain Healthcare. Governance for quality in a high performance system)
2015	Health Care Analysis	*Vårdval och jämlik vård inom primärvården* (Choices and equity in primary care)
2016	The Government of Sweden	SOU 2016:2: *Effektiv vård* (Efficient health care)
2016	The Swedish Agency for Public Management	Samlad uppföljning av den statliga styrningen av kommuner och landsting (*Follow-up of public management of municipalities and county councils*)
2017	The Government of Sweden	SOU 2017:56: *Jakten på den perfekta ersättningsmodellen* (In pursuit of a perfect reimbursement model)
2017	The Government of Sweden	SOU 2017:48: *Kunskapsbaserad och jämlik vård* (Knowledge-based and equal care)
2017	SALAR	*Debatt pågår! Offentlighetens organisering* (An ongoing debate! Organizing public service)
2017	SALAR	*Debatt pågår! Styrning och professionellt inflytande i offentliga organisationer* (An ongoing debate! Management and professional influence in public organizations)
2017	Ministry of Health and Social Affairs	*SBU:s kartläggning av kunskapsläget kring värdebaserad vård* (SBU’s mapping of knowledge on value-based care)
2017	Health Care Analysis	*Primärvården i belysning. Jämförelser mellan landsting och regioner 2011–2015* (Primary care highlighted. Comparisons between county councils and regions 2011–2015)
2017	The Norrbotten Region	*Primärvården i fokus* (Primary care in focus)
2018	The Government of Sweden	SOU 2018:47: *Med tillit växer handlingsutrymmet – tillitsbaserad styrning och ledning av välfärdssektorn* (Trust increases room for manoeuvre)
2018	The Government of Sweden	SOU 2018:55: *Styrning och vårdkonsumtion ur ett jämlikhetsperspektiv* (Governance and health care consumption from an equity perspective)
2018	SALAR	Vem kör egentligen? (Who’s driving, actually?)
2019	The Government of Sweden	SOU 2019:42: *Digifysiskt vårdval* (Digi-physical choices in health care)
2019	The Government of Sweden	SOU 2019:43: *Med tillit följer bättre resultat* (With trust comes better results)
2019	The Ministry of Health and Social Affairs	*Styrmodeller i hälso- och sjukvården – förslag till modell för etisk analys* (Models for governance in health care – suggestion of a model for ethical analysis)
2019	The Ministry of Health and Social Affairs	*Värdebaserad vård* (Value-based care)
2019	Forum for Health Policy	*Vem styr hälso- och sjukvården*? (Who is managing the health care sector?)
2020	The Government of Sweden	SOU 2020:15: *Strukturförändring och investering i hälso- och sjukvården – lärdomar från exemplet NKS* (Structural changes and investments in health care – lessons learned from the example of NKS)

### Analysis

For the descriptive overview of all 106 reports, a matrix was constructed, based on the above-mentioned research questions.

The 36 selected reports were analysed using inductive content analysis [18]. Our aim was not to organize that data from concepts that were chosen in advance but to do an unbiased review of the reports based on the research questions, hence an inductive approach was chosen. First, all selected reports (*n* = 36) were read thoroughly and a summary of each report was written. These summaries resulted in about 140 pages of new, condensed text. The next step consisted of coding the condensed text. The text was read through several times and codes answering the research questions were identified. Thereafter, quotes illustrating each code were collected from the reports. Finally, the codes were sorted into categories, answering the research questions. A description of the analysis process is found in Table 2.

**Table 2:** Description of the analysis process

Meaning unit	Condensed meaning unit	Code	Category
“Two starting-points have been established for the group’s work (…) the governance models should be compatible with two principles: equity in health care and public financing.”	Governance models compatible with equity in health care and public financing	Views on equity	Equity as geographical sameness
“The goal is to investigate how the government can work for a sustainable system for health care, focusing on promoting health and preventing illness (…) with the goal of equity in health all over the country.”	The goal is equity in health all over the country	Views on equity	Equity as geographical sameness
“The value of care is created in the encounter and interaction between the patient and the health care system.”	Value is created in the encounter with the patient	Value-based care	Efficiency and value-based care
“The goal is to suggest increased compliance to national guidelines, in order to achieve a knowledge-based and equal health care.”	Striving for knowledge-based care	Knowledge-based care and governance	Knowledge-based management
“A structure for national knowledge-based management is needed.”	Striving for knowledge-based health care management	Knowledge-based care and governance	Knowledge-based management
“Increased trust in governance can contribute to increased room for maneuver in the encounter between citizens and professionals.”	Increased trust improves the encounter between citizens and professionals	Increased trust in professionals’ competence	Management based on trust
“Trust can be seen as a management principle, where co-operation is the base and the professional competence central.”	Trust is a management principle, where professional competence is central,	Increased trust in professionals’ competence	Management based on trust

## Results

### Descriptive mapping

**Fig. 1:**
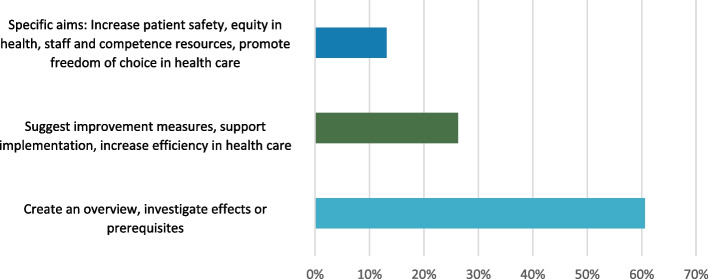
Overview of the aims of the studied reports

**Fig. 2:**
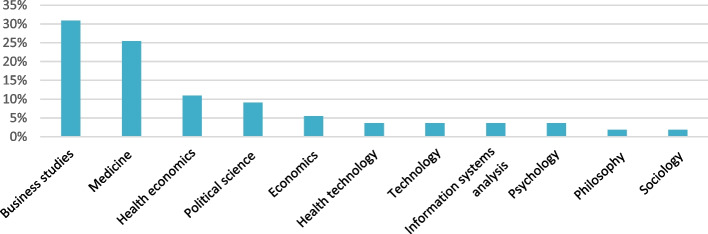
Disciplines academic experts represent in the studied commissioned reports

**Fig. 3:**
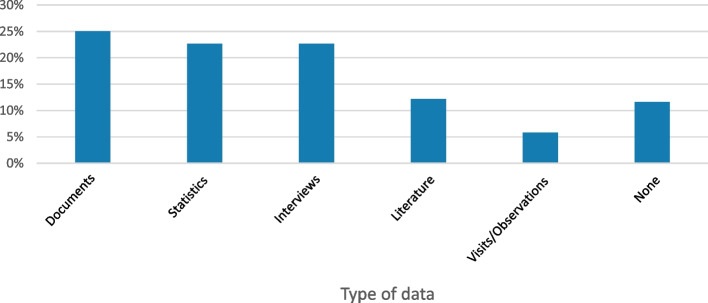
Types of data the studied commissioned reports built on

The mapping of all 106 reports, published from 1993 to 2020, showed that a wide range of reports were commissioned during this time. Many of the reports had very general and vague purposes (such as providing an overview), while a few had more specific aims, such as investigating effects and consequences of new control models or suggesting improvement measures (Fig. 1). Academic experts involved in creating the reports often represented economics or business studies. Political scientists and medical researchers were represented in a minority of the reports. Only in rare cases did the academic experts come from psychology or sociology (Fig. 2). A majority of the reports built on previous data in the form of statistics or interviews. Only 5% of the reports based their arguments on direct observations of healthcare practices (Fig. 3).

A more general observation is that the main tendency in the reports shifted over time, from providing input regarding specific administrative *problems* to acting as support for specific administrative *solutions* (such as “value-based care” or “knowledge management”). Hence, over time the reports appeared to be less independent and more oriented towards particular predetermined policy orientations and directions.

The mapping also showed that several of the reports were characterized by conflicting intentions or principles. For instance, the pursuit of standardization embodied in knowledge management that some reports argue for might come into conflict with the professional context-dependent judgements, person-centredness and patient influence prescribed in other documents. In several cases, the scientific data requested from academic experts was not used in the reports’ conclusions and recommendations; data was selected, and relevant information and knowledge for the decision to be made thus seemed to be neglected.

### Content analysis

The content analysis resulted in four categories: *Equity as geographical sameness, Efficiency and value-based care, Knowledge-based management* and *Management based on trust.* In the following, each category is described and exemplified with quotations from the studied reports.

### Equity as geographical sameness

The first category concerns the theoretical and ideological starting points in the analysed reports. We found that reports from the 1990s and the beginning of the twenty-first century often based their arguments on ethical assumptions mirrored in the guidelines for priority setting, established by the Swedish Parliament in 1997 [17]. Reports from this period therefore emphasized equity in healthcare, human dignity and care provision based on medical needs. The ethical platform for priority setting in Swedish healthcare is described in a report from the National Delegation for Priority Setting [19]. The Delegation was formed in 1998. Their task was to follow up the parliamentary decision on guidelines for priority setting in Swedish healthcare, based on the ethical principles of human dignity, need and solidarity and cost-efficiency. The Delegation’s assignment was to spread information and knowledge about the guidelines, initiate discussions about them and develop new methods for controls and follow-ups of the guidelines.

Also, reports written before the parliamentary decision on priority setting prescribe equity in health based on prioritization according to medical needs. This was the case in a report produced by a group of experts assigned to a public inquiry on Swedish healthcare for the twenty-first century (often referred to as “HSU 2000”) [20]. Here, the authors declare that their investigation starts from two ground rules, namely *equity in health* and *public financing of healthcare* [20, p. 11]. These are highly ethical starting points, based on the ethical platform for priority setting, primarily the principles of human dignity and need and solidarity.

Likewise, the final report from the public inquiry on Swedish healthcare for the twenty-first century (“HSU 2000”) subscribes to what the authors call “the national goals and ethical principles for Swedish healthcare”, that is, equity in health, respect for human dignity and equal value and, finally, priorities based on needs [21]. At the same time, the report declares: “The committee is aware that these goals will never be fully achieved” [21, p. 54].

In this report, the authors openly refer to the parliamentary public inquiry concerning priority setting in healthcare [17] and emphasize that the political discussion on priorities in healthcare must be a long-term and ongoing process. At the same time, the authors declare that value changes in society can influence the healthcare organization. For example, they mention new forms for financing healthcare provision, such as more private caregivers alongside the hitherto mostly tax-funded healthcare in Sweden. This can increase citizens’ freedom of choice in healthcare, but it can also contribute to inequality in health. The authors conclude:

According to the committee, increased freedom of choice in healthcare is desirable, but it must not be implemented at the expense of the national goals for healthcare. /---/ Municipalities and county councils are responsible for the quality of care, including activities performed by private actors [21, p. 69].

These worries led to the conclusion that the government must ensure that care is provided in line with the national goals and ethical principles for priorities through economic efforts [21, p. 82–83]. Hence, the authors of this report clearly identify the individuals or authorities to whom they assign responsibility for their suggestions.

These reports reflect the trend of equity in healthcare found in documents from the 1990s and the beginning of the twenty-first century. A reasonable interpretation is that “equity in health” in these cases refers both to *equity in quality of care* and *equity in access to care*. However, a shift of focus can be found in reports from 2010 and onwards. In a report entitled *Make it easier!*, the task for the investigator was to:

…investigate how the government can work for a sustainable system for healthcare, focusing on promoting health and preventing illness (…) with the goal of equity in health *all over the country* [22, p. 13; emphasis added].

Here, equity in healthcare is mentioned, but a slight difference in meaning can be found compared to the previous reports [20, 21], as the words “all over the country” have been added. The same pattern can be found in several reports from this period, namely, that equity in health has come to mean primarily geographical sameness, i.e., prescribing that the quality of treatment and care must not suffer from geographic variation. This can be interpreted as a slight shift from previous definitions, where equal care was instead defined in terms of equity in human value and rights, and thereby emphasizing not only equity in quality of care but also equity in access to care. We argue, that this is an important ethical difference, as it is possible that the treatment of myocardial infarction, for example, is the same all over the country based on standardized clinical practice guidelines, but access to care might still not be equal between people from different socio-economic groups or ethnic minorities. Thus, it can be concluded that the definition of equity in healthcare has changed over time in the studied reports, from equity in values and rights between persons or groups to a focus on equity as geographical sameness, achieved through following the same evidence-based medical guidelines; thereby emphasizing equity in quality of care at the expense of equity in access to care.

### Efficiency and value-based care

The second category describes how several reports argued for efficiency in healthcare and for value-based care. One example of this is a report from the Swedish Association of Local Authorities and Regions (SALAR), published in 2014. The report is an investigation of Intermountain Healthcare in Salt Lake City in Utah, USA. The goal was to study an efficient healthcare system, which Sweden could learn from. Inspired by Intermountain Healthcare, the report emphasizes value-based care and efficiency in the healthcare system at the expense of ethical values such as equity and need [23].

A similar pattern is found in a public inquiry from 2016, with the timely title *Efficient healthcare* [24]. This report makes frequent references to “value-based care”. For example, it says that “the value of care is created in the encounter and interaction between the patient and the healthcare system” [24, p. 18]. Therefore, the author argues, the patient must be given extended agency and be allowed to participate more in the process around his/her care.

Such an argument is well in line with the definition of value-based care found in a report ordered by the Ministry of Social Affairs and produced by the foundation Leading Healthcare [25]. It discusses how value-based care was for some time described as the universal solution for all of the problems that healthcare systems in the West were facing [25]. The definition of value-based care stems from Michael Porter and Elizabeth Teisberg, both from Harvard Business School in Boston. In the beginning, value-based care was referred to as “value-based competition”. According to Porter and Teisberg, it is calculated according to the following formula: *Value* = *effect/cost.*

Krohwinkel and co-workers argue that this formula relates to organizational theory’s concept of efficiency, which describes an organization’s ability to transform resources into products or services. “Efficiency is described as the extent to which the goals are fulfilled in relation to the use of resources”, the authors state [25, p. 31]. However, this implies that healthcare is provided in a way that can be measured and compared.

Such views are also found in other commissioned reports, including the above-mentioned report *Efficient healthcare*. This report defined efficiency in healthcare as providing “the most and the best to the patient, given the resources at hand” [24, p. 19]. Apart from that, the report states that the patient should be a “co-producer” of his/her care – a concept that is, however, poorly defined in the report.

### Knowledge-based management

A third trend found in the analysis concerned an increased focus on knowledge-based governance in the reports. Examples of this trend are already evident in reports from the beginning of the twenty-first century. For example, the SALAR report “Knowledge-based management, governance and development in healthcare” discusses this [26]. A starting point in the report is to ensure the development of knowledge in the field of healthcare governance. Based on three models of governance found in political science – hierarchy, market and network – the authors suggest an increased focus on knowledge-seeking in healthcare governance and increased research on knowledge-based healthcare governance.

The discussion on knowledge-based governance is closely related to the focus on geographical sameness discussed above. SOU 2017:48, *Knowledge-based and equal healthcare*, shows this in the title. The aim of that report was to suggest means to achieve increased compliance with national clinical guidelines for treatments and therapies. Through increased compliance, knowledge-based management will be achieved, and that will also improve equity in healthcare, the authors argue [27]. Better compliance with national guidelines was suggested earlier in SOU 2016:2 (*Efficient healthcare)* as described above [24], but according to SOU 2017:48, this has not been achieved, which is why the same message is repeated again [27].

At the beginning of the report SOU 2017:48 it is stated that every patient encounter should be based on “the best possible knowledge” [27, p. 17]. But what knowledge is the report referring to in this case? The goal of equity in healthcare is mainly an ethical goal, but it becomes clear that the knowledge referred to in this report is evidence-based medical knowledge. For example, the authors refer to national clinical care programmes and guidelines. In spite of such national guidelines and regulations, the authors state, there are still inequities in health between women and men and between different parts of the country [27, p. 18]. This passage shows that the authors of the report embrace a definition of equity similar to that in SOU 2016:2 [24], namely, that equity is about geographical sameness. However, they enlarge the definition by adding that it also concerns equity between women and men.

A more critical perspective when it comes to knowledge-based governance is found in a report written by Karin Fernler in 2012. The aim of this report was to investigate the possibilities of introducing evidence-based management and governance in the healthcare system. In medicine, evidence-based knowledge is founded on a common base of knowledge with general validity over time, independent of context. The main question in Fernler’s report is whether it is also possible to find such a common base of knowledge when it comes to healthcare governance [5].

Evidence-based knowledge in general has been defined as the “best available scientific knowledge” [5, p. 79]. Fernler adheres to this definition and argues for a strong emphasis on scientific knowledge also in knowledge-based governance. However, two critical points are raised, namely, that decision processes in healthcare governance are seldom goal rational and that it is difficult to formulate a stable base of knowledge from organizational theory. Fernler also points to the fact that organizations often follow fashion when it comes to management [5, p. 82]. Further, she lists the following aspects of organizational theory, which can make knowledge-based governance difficult:

- The theory includes several vague and changeable concepts.
- It is characterized by a variety of theoretical perspectives and a variety of contexts.
- It depends on a critical balancing of generalizable and relevant knowledge.

Therefore, Fernler’s conclusion is that evidence-based knowledge is hard to find for healthcare governance. She writes:

To govern practice-based priority setting and organization of healthcare based on knowledge requires that one considers a wide variety of perspectives and claims of knowledge, which might come into conflict with one another [5, p. 91].

In spite of this critical report, several public inquiries and other reports in our studied material continue to argue for knowledge-based governance. However, the reports that argue for this form of governance seem to refer to evidence-based *medical* knowledge, not knowledge about management and governance. This was found, for example, in SOU 2017:48, *Knowledge-based and equal healthcare* [27] and SOU 2016:2, *Efficient healthcare* [24].

### Management based on trust

So far, we have seen that reports from the 1990s reflected a strive for political sameness and thereby adhered to the ethical guidelines for priority setting, focusing on human dignity, solidarity and equity. At the beginning of the twenty-first century, different versions of value-based care were put forward in the commissioned reports, and cost-control and efficiency became dominant values. The third category concerned the trend of knowledge-based management and the debate around this concept when it comes to healthcare governance. However, the analysis also revealed a fourth category, namely, governance based on trust. This trend can be interpreted as a reaction to the emphasis on detailed control, cost-efficiency and measurable goals that dominated the reports commissioned after 2010.

The focus on governance based on trust is evident in that a special committee was formed around this concept in 2016. A public inquiry was assigned and resulted in three reports: SOU 2017:56 (*In pursuit of a perfect reimbursement model*) [28], SOU 2018:47 (*Trust increases room for manoeuvre*) [29] and SOU 2019:43 (*With trust comes better results*) [30]. The aim was to analyse forms of governance in the public sector that consider the professionals’ competence and experience. An important aspect of this work was to investigate the effect of different reimbursement systems in the healthcare sector.

Unlike many other reports in our material, this one relied on input from researchers and academics in order to have a critical perspective on the work. However, it is hard to see in the reports where, how and to what extent these contributions have been used. Concerning the analysis of reimbursement systems, SOU 2017:56 argues that the current systems are so administratively complicated that they hinder the professionals from working with their main tasks (providing care). This can undermine professionals’ ability to follow ethical guidelines, the authors argue [28, p. 180]. The committee argues for a form of trust-based governance, which they define as governance that has no unnecessary controls and that takes advantage of the professionals’ competence. It is argued that this will lead to better quality for patients and citizens. Further, economic governance must be replaced by other forms of governance, according to the authors, based on dialogue and communication [28].

The main report from the committee on trust in healthcare governance is SOU 2018:47 (*Trust increases room for manoeuvre*) [29]. Although the focus is on criticizing the trend to neglect healthcare professionals’ competence and experience, this report starts with an assumption that resembles assumptions in reports on value-based care, namely, that value and quality are created in the encounter between healthcare personnel and the patient [29, p. 16 and 49ff]. As was the case in previous reports that reasoned in the same way, this is a statement that is vague and poorly defined. Obstacles to a value-building encounter, according to SOU 2018:47, include lack of competence development and lack of learning opportunities for the staff. But is the report referring to ethical, aesthetic or economic values? The text is not very clear on this. Also, the core concept, “trust”, can be defined in different ways and it is not evident what sort of trust the reports are really arguing for in this case.

## Discussion

The aim of the present study was to conduct an overview and a content analysis of reports on Swedish healthcare governance commissioned by national and regional authorities between 1993 and 2020. In total, 106 reports were collected and analysed in a descriptive overview. In a strategic sample, 36 of these reports were selected for an in-depth content analysis.

### Descriptive mapping

The mapping of the 106 reports showed that the aim of the reports varied from giving an overview and investigating effects and consequences of new control models to more concrete goals, such as suggesting improvement measures. Experts involved in the producing of reports were in most cases from economics or business studies. It is therefore not surprising that economic perspectives had an advantage over, for example, ethical reasoning in the reports. This is notable, as both law [16] and ethical guidelines [19] in Swedish healthcare emphasize ethical values. The analysis also revealed that data seemed to be used selectively in that the input that was sometimes requested from academic experts was not always used in the reports’ conclusions and recommendations.

Only a few of the investigated reports were based on observations or empirical data. Further, we found that several reports were characterized by conflicting intentions or principles, often between them but in some cases also within the same report. One example was the conflict between the pursuit of standardization (for example, in the form of national clinical guidelines) on the one hand and professional context-dependent judgements based on person-centredness and patient influence on the other. Arguments for both of these values were put forward in, for example, the commissioned reports on governance based on trust [28–30].

The great number of reports can be seen as examples of *informative governance* that Swedish healthcare governance has increasingly begun to rely upon [31]. Informative governance is characterized by progress towards evidence-based policymaking, inspired by evidence-based medicine and clinical practice guidelines in medical decision-making [31]. This also supports the findings from Feldman and March [4] and Fernler [5], who have all argued that organizations often collect more information than they can use. A possible explanation for this overload of expert reports is that authorities and organizations want to provide an image of rationality behind their decisions on new governance forms. Further, the great number of commissioned reports can, as has been argued by Ahlbäck Öberg and Öberg, become a means to provide legitimacy to the authorities’ decisions [3].

### Content analysis

The in-depth content analysis of 36 commissioned reports resulted in four categories, *Equity as geographical sameness, Efficiency and value-based care, Knowledge-based management* and *Management based on trust.*

The first category, *Equity as geographical sameness*, showed how the definition of equity in healthcare had changed during the studied period. The early reports in our material, published in the 1990s or at the beginning of the twenty-first century, adhered to the Swedish principles of priority setting from the 1990s, namely, *human dignity, need and solidarity* and *cost-efficiency* [17, 19]. The focus in the reports was on the first two principles – human dignity and need and solidarity – as they emphasized equity in health for the whole population.

The three principles for priority setting relate to central ethical concepts, such as autonomy, justice and the proportion between efforts and effects [32]. They also reflect the long legal and ethical effort to achieve equity and equality in Swedish healthcare. Based on the principle of human dignity, *equity* would mean that all persons have the same human rights and are entitled to have these rights respected. Hence, equity in this sense applied to the healthcare system is about securing access to healthcare and treatment according to medical needs for everyone. *Equality* can be understood as a concept that prescribes every human’s right to equal opportunities and treatment [33].

Our results showed that the expert reports commissioned at the beginning of the studied period adhered to a classic definition of equity, namely, that all persons have the same value and human rights and that they are therefore entitled to have these rights equally respected [33]. Further, the principle of need and solidarity that is found in the Swedish guidelines for priority setting, which the early reports in our study referred to, prescribes special attention to the least advantaged. This can be interpreted as in line with the difference principle developed by John Rawls in his classic work on justice [33].

However, reports published after 2010 presented a slightly different understanding of equity, that is, equity as geographical sameness. That was the case in SOU 2016:2 [24] and also in SOU 2017:48 [27]. In the latter report, the definition was broadened to include equity between women and men in healthcare. Although it is positive that this report enlarged the definition of equity in healthcare, from focus on geographical sameness to a definition that also included equity between women and men, it still left out many factors related to equity and equal opportunities and treatment, such as ethnicity, socio-economic status, education, dis/ability and age. It is possible to argue that geographical sameness is an important aspect of the Rawlsian understanding of equity mentioned above, but this result is still an example of how the understanding of equity in the studied reports has been narrowed down during the studied period of time.

We argue that, by allowing the principle of equity to primarily denote geographical sameness, the decision-makers deviate from the statutory ethical platform. Instead of focusing on equity in its traditional form, reports commissioned after 2010 emphasize equity in the form of following evidence-based clinical guidelines all over the country. A reasonable interpretation of this development is that more focus over time has been put on *equity in quality of care*, at the expense of *equity in access to care*. Although it is possible to argue that equity of access as a principle might be integral to institutional and funding arrangements, we still argue that both aspects of equity (i.e. equity in quality of care and equity in access to care) need to be explicitly spelled out in commissioned reports, in order to provide a sufficient argumentation about equity in healthcare.

The described development can be interpreted as an example of increased *standardization* in healthcare, which is a trend not only in Sweden but in many healthcare systems. Previous research has shown how evidence-based clinical guidelines are used to establish alignment in the treatment of patients in several European countries [31, 34, 35]. That increased standardization can conflict with the healthcare provider’s ambition to give individualized care has previously been pointed out [31]. The ethical problem with this development is that it might limit professional autonomy in healthcare providers as well as reduce respect for patient expectations as the focus is on following national clinical guidelines.

The described development can be interpreted as an increased focus on economy and cost-effectiveness at the expense of other ethical values such as equity and equality in healthcare. This was further described in the second category: *Efficiency and value-based care*. The definition of value-based care stems from Michael Porter and Elizabeth Teisberg, both from Harvard Business School in Boston [36]. In the beginning, value-based care was referred to as “value-based competition”. According to Porter and Teisberg, it is calculated according to the following formula: Value = effect/cost [36]. It can be argued that the meaning of this formula is rather unclear, but the bottom line is that value is understood in economic terms, not as an ethical value. As argued by Krohwinkel et al. [25], this implies a quite narrow view on what the healthcare system should strive for “as only one dimension of efficiency” is considered [25]. Central ethical values such as equity and equality are thereby left out in the model of value-based care.

The third category that was developed from our material, *Knowledge-based management,* can also be understood as a form of standardization. Here, the emphasis on compliance with national guidelines was even more evident. The problem with this trend, however, is that it is not clear what kind of knowledge the reports refer to. Our analysis revealed that the commissioned reports mainly referred to national guidelines for clinical practice, not knowledge about management or governance as found in organizational theory. The reason behind this might be that evidence-based knowledge cannot be used for management and governance in the same way as it can, for example, in medical treatments. According to Fernler [5], decision processes in healthcare governance are seldom goal rational and it is difficult to formulate a stable base of knowledge from organizational theory. Rather, organizational theory includes several vague and changeable concepts as well as a variety of theoretical perspectives. All these aspects make evidence-based knowledge about healthcare governance hard to establish.

The last category, *Management based on trust*, can be seen as an attempt to respond to the problems described above, developed due to increased standardization. As requirements to follow national clinical guidelines can conflict with professional autonomy, the trend towards increased trust in professionals’ competence and experience seems logical. Governance based on trust is described in the reports as a system free from unnecessary controls and a situation where economic governance is combined with other forms of governance, based on dialogue and communication [27–29]. However, the fact that arguments for both standardization and trust can be found in commissioned reports on Swedish healthcare governance at the same time can arguably create tensions. This also raises questions. How should the system be enabled to combine these different control models? Is it even possible to combine them? The analysis revealed that such value conflicts were not recognised in the reports and therefore not dealt with.

An important finding in our investigation is that the commissioned reports often mirrored the context in which they were produced. In this case, our results support the findings of Svallfors and co-workers [37] and Larsson [6], who found that organizations often follow trends and “fashions” for governance during a certain time. This runs the risk of creating governance systems that are poorly investigated and lack consequence analysis before being launched.

In the light of the statutory ethical goals in Swedish healthcare, the results from the in-depth analysis seem quite problematic, in the sense that the ethical underpinnings in healthcare governance tend to lose their meaning and deviate from the law’s requirements. This is primarily relevant concerning the use of equity in a new sense (geographical sameness). It risks shifting attention from people to procedures and deviate from the law’s requirement for equity in care for the whole population. A policy implication of our results is thus that new control models in healthcare need to consider both aspects of equity; i.e., geographical sameness as well as equal opportunities for individuals and groups.

## Conclusion

Our study revealed how the purpose of commissioned reports on Swedish healthcare governance had shifted over time, from providing input on administrative problems to supporting specific administrative solutions. This is shown in the broad consensus on value-based care and knowledge-based management found in the studied reports. Academic experts involved in the producing of reports were in most cases from economics or business studies, which might explain the focus on economy and control models in the materiel.

The reports could also express conflicting values and goals, for example, simultaneously arguing for standardization – which can conflict with professional autonomy – and trust in professional competence – which emphasizes professional autonomy. Further, the analysis showed how the definition of equity in care had changed, from a focus on equity in access to care in the reports produced in the 1990s to an emphasis of arguments for geographical sameness and equity in quality of care in the later reports.

The great number of reports implies that the system risks requesting more information than it can handle. Further, it might result in reports where the same message is repeated in different documents, or – perhaps an even bigger problem – it might create conflicts of interest and value tensions between what is suggested in different reports. In sum, our analysis showed two dominant trends in the analysed reports, namely, increased standardization and arguments for trust in the system.

Commissioned reports can have substantial consequences for new reforms of management practices in healthcare. It is therefore important to investigate them critically. The results of our investigation may contribute to a more comprehensive and adequate model for acquiring and using expert reports regarding healthcare governance, both in Sweden and in similar healthcare systems.

## Data Availability

The dataset used and analysed during the current study is available from the corresponding author on reasonable request.
